# Supplementary material for: Performance assessment of total RNA sequencing of human biofluids and extracellular vesicles
Source: Sci Rep. 2019 Nov 26;9:17574. doi: 10.1038/s41598-019-53892-x (PMC6879519; doi:10.1038/s41598-019-53892-x)
Supplement: Supplementary file 1 — Supplemental Figures [file 41598_2019_53892_MOESM1_ESM.pdf]

Performance assessment of total RNA sequencing of human biofluids and extracellular vesicles

Celine Everaert [1,2,†], Hetty Helsmoortel [1,2,†], Anneleen Decock [1,2], Eva Hulstaert [1,2,3], Ruben Van Paemel [1,2], Kimberly Verniers [1,2], Justine Nuytens [1,2], Jasper Anckaert [1,2], Nele Nijs [4], Joeri Tulkens [2,5], Bert Dhondt [2,5,6,], An Hendrix [2,5], Pieter Mestdagh [1,2] and Jo Vandesompele [1,2,4*]

[1] Center for Medical Genetics, Department of Biomolecular Medicine, Ghent University, Ghent, Belgium
[2] Cancer Research Institute Ghent, Ghent, Belgium
[3] Department of Dermatology, Ghent University Hospital, Ghent, Belgium
[4] Biogazelle, Zwijnaarde, Belgium
[5] Laboratory of Experimental Cancer Research, Department of Human Structure and Repair, Ghent University, Ghent, Belgium
[6] Department of Urology, Ghent University Hospital, Ghent, Belgium

† These authors contributed equally to this work

* Correspondence: jo.vandesompele@ugent.be; Tel.: +32 9 332 1381

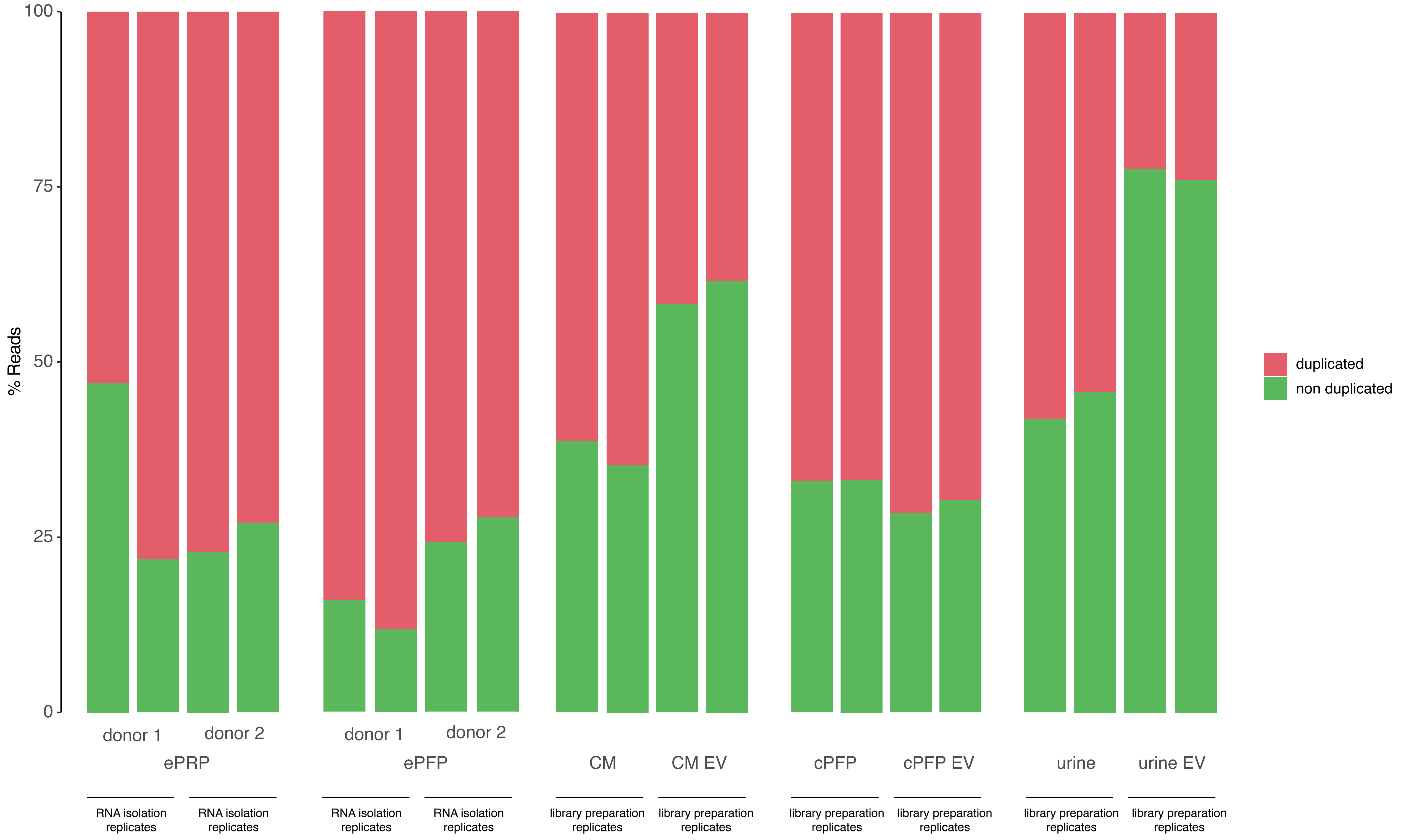

Supplemental Figure 1 Read duplication levels are markedly different among different biomaterials.

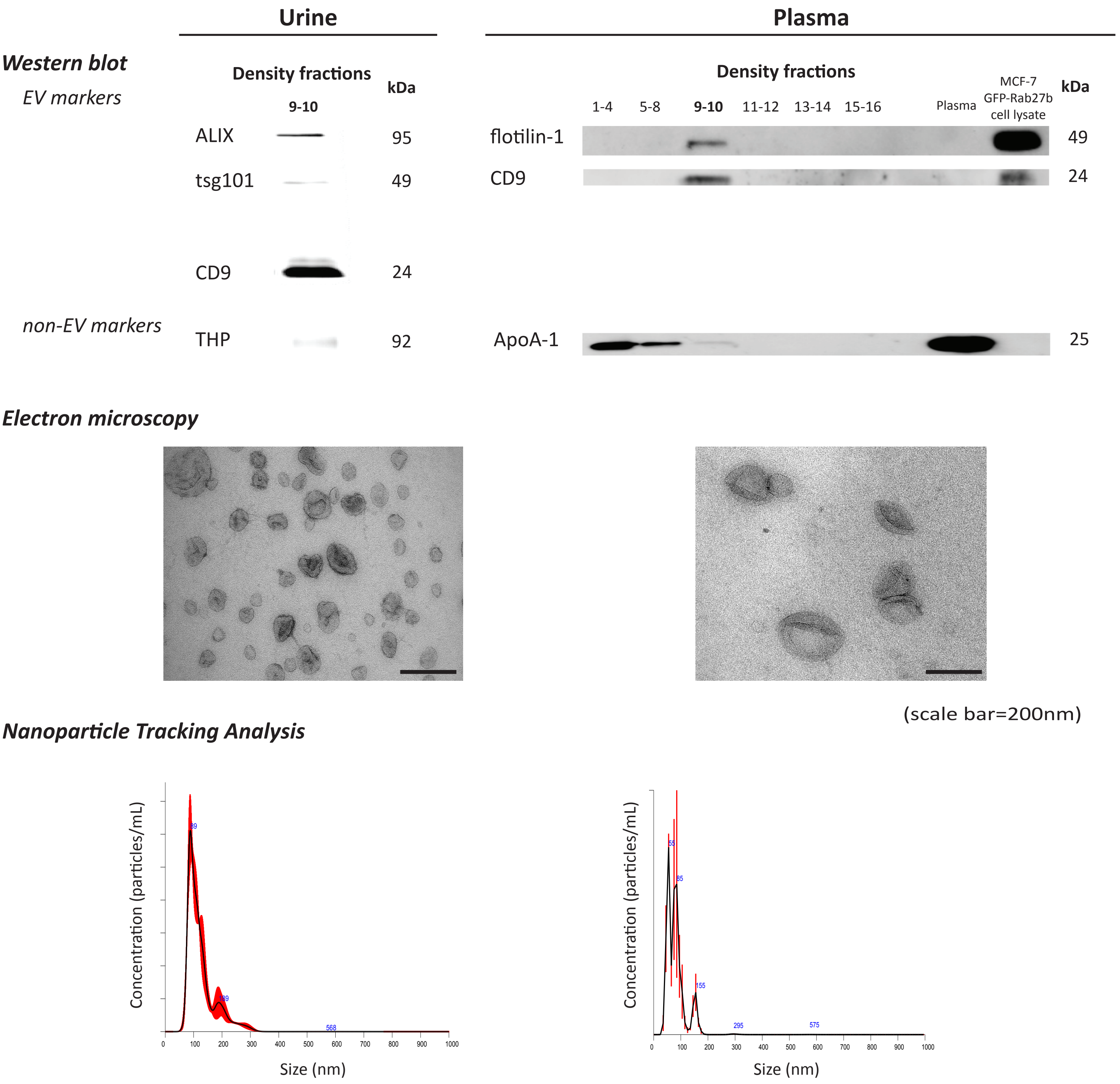

Supplemental Figure 2a Characterization of EV from urine and plasma samples. Proteins are analyzed by performing western blot using specific EV markers (ALIX, tsg101, CD9 and flotillin-1) and non-EV markers (THP and ApoA-1). Results were obtained by running four different SDS-PAGE gels and full-length blots are presented in Supplementary Figure 12. EV samples (density gradient fractions 9-10) are enriched in EV proteins and depleted for contaminants. EVs were qualitatively and quantitatively analyzed by electron microscopy and nanoparticle tracking analysis.

MCF-7 GFP-Rab27b

Western blot

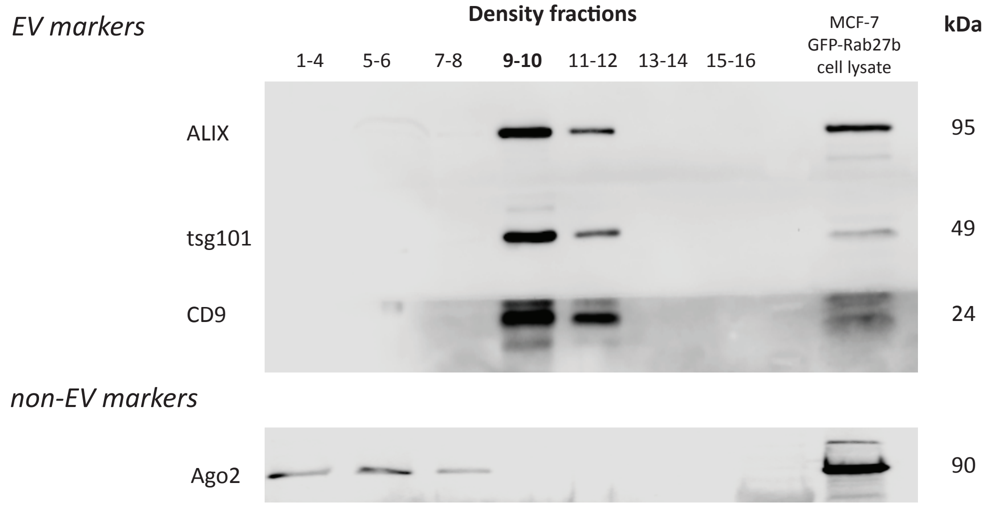

Electron microscopy

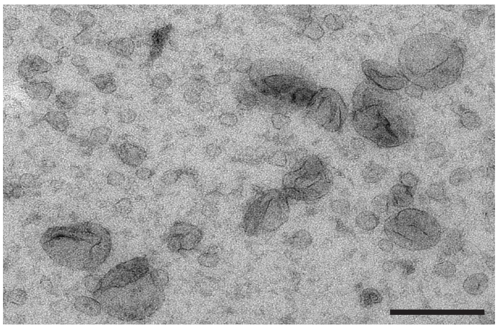

(scale bar=200nm)

Nanoparticle Tracking Analysis

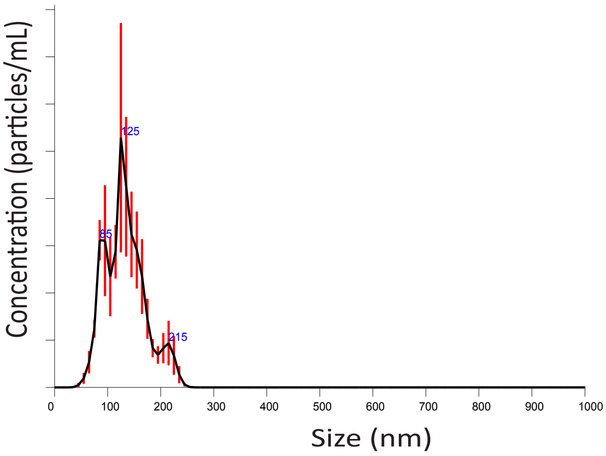

Supplemental Figure 2b Characterization of EV from MCF-7 GFP-Rab27b cells. Proteins are analyzed by performing western blot using specific EV markers (ALIX, tsg101 and CD9) and non-EV markers (Ago2). Results were obtained by running two different SDS-PAGE gels and full-length blots are presented in Supplementary Figure 12. EV samples (density gradient fractions 9-10) are enriched in EV proteins and depleted for contaminants. EVs were qualitatively and quantitatively analyzed by electron microscopy and nanoparticle tracking analysis.

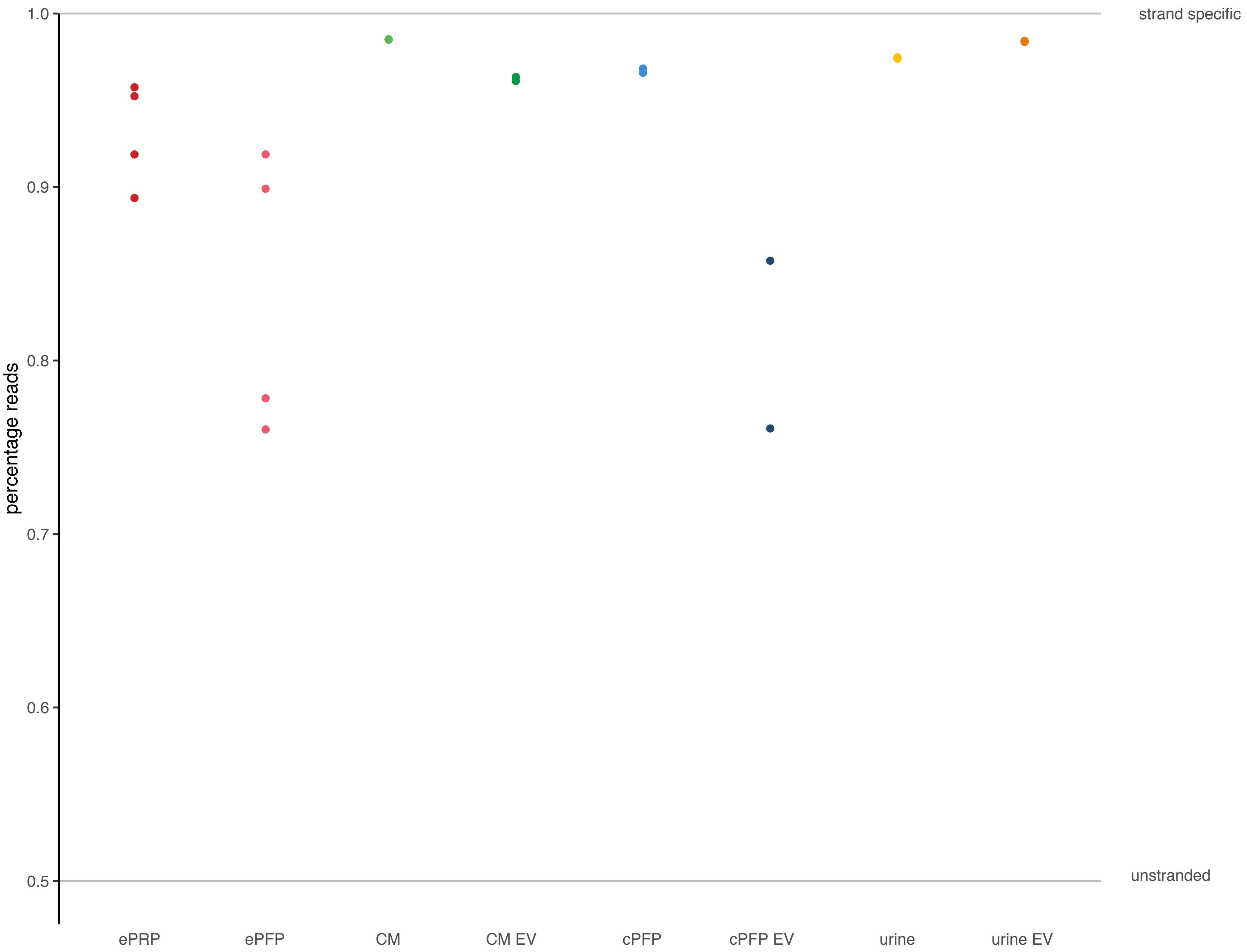

Supplemental Figure 3. Percentage of reads originating from the sense strand to demonstrate good strandedness of the kit.

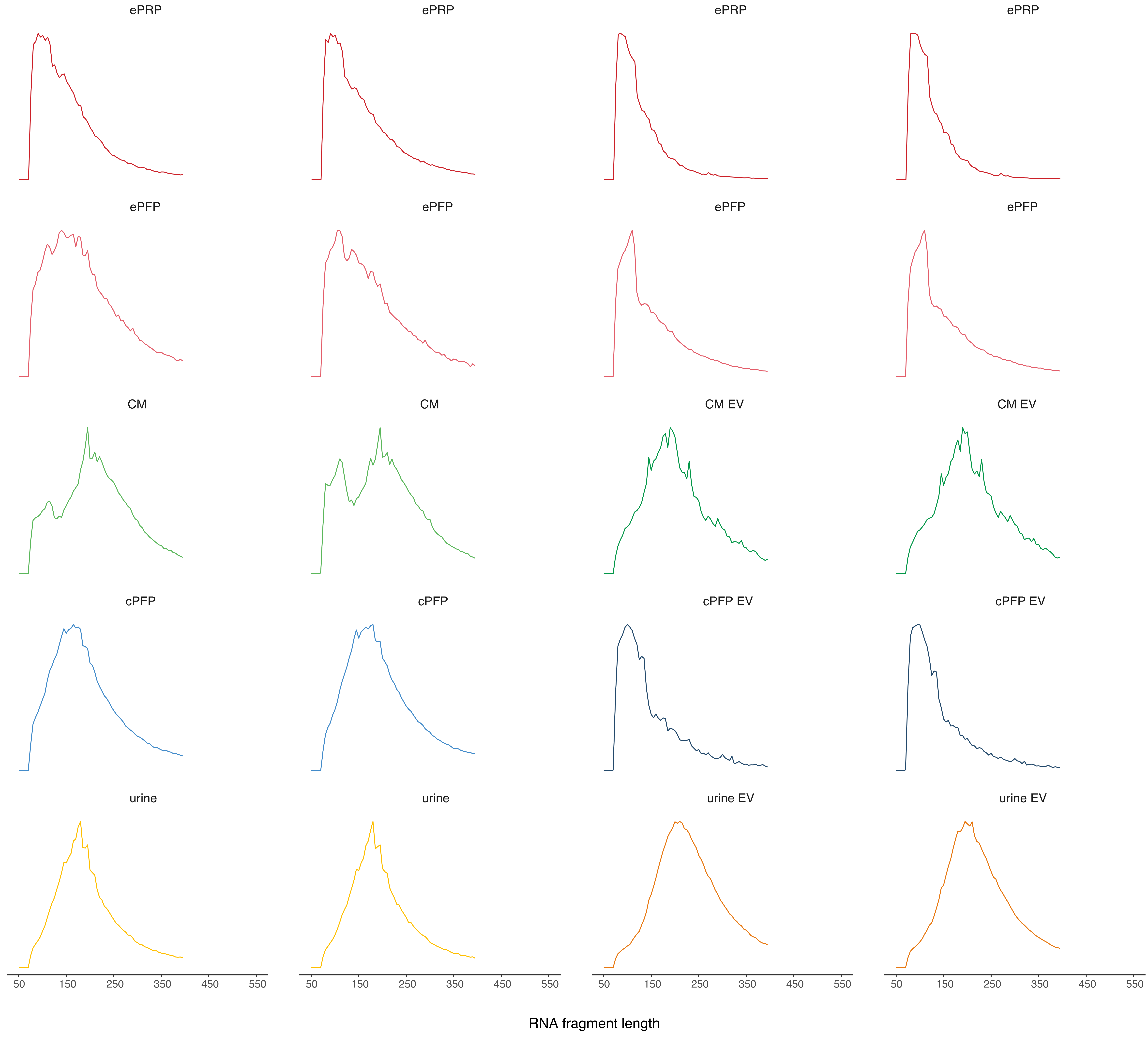

Supplemental Figure 4 RNA fragment size distribution shows shorter lengths in plasma derived libraries.

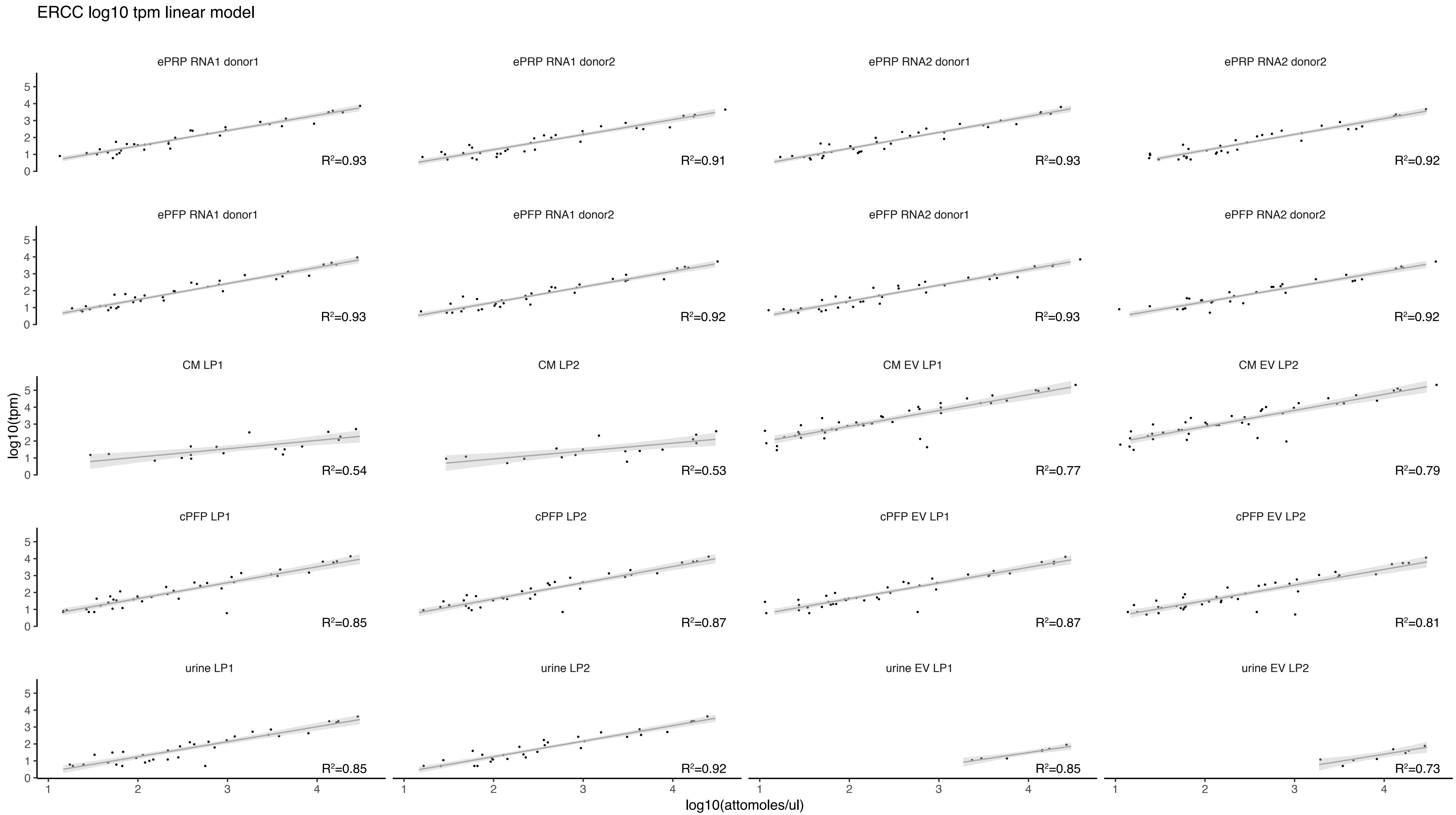

Supplemental Figure 5 Good concordance between expected concentrations and observed TPMs. LP = library prep replicate.

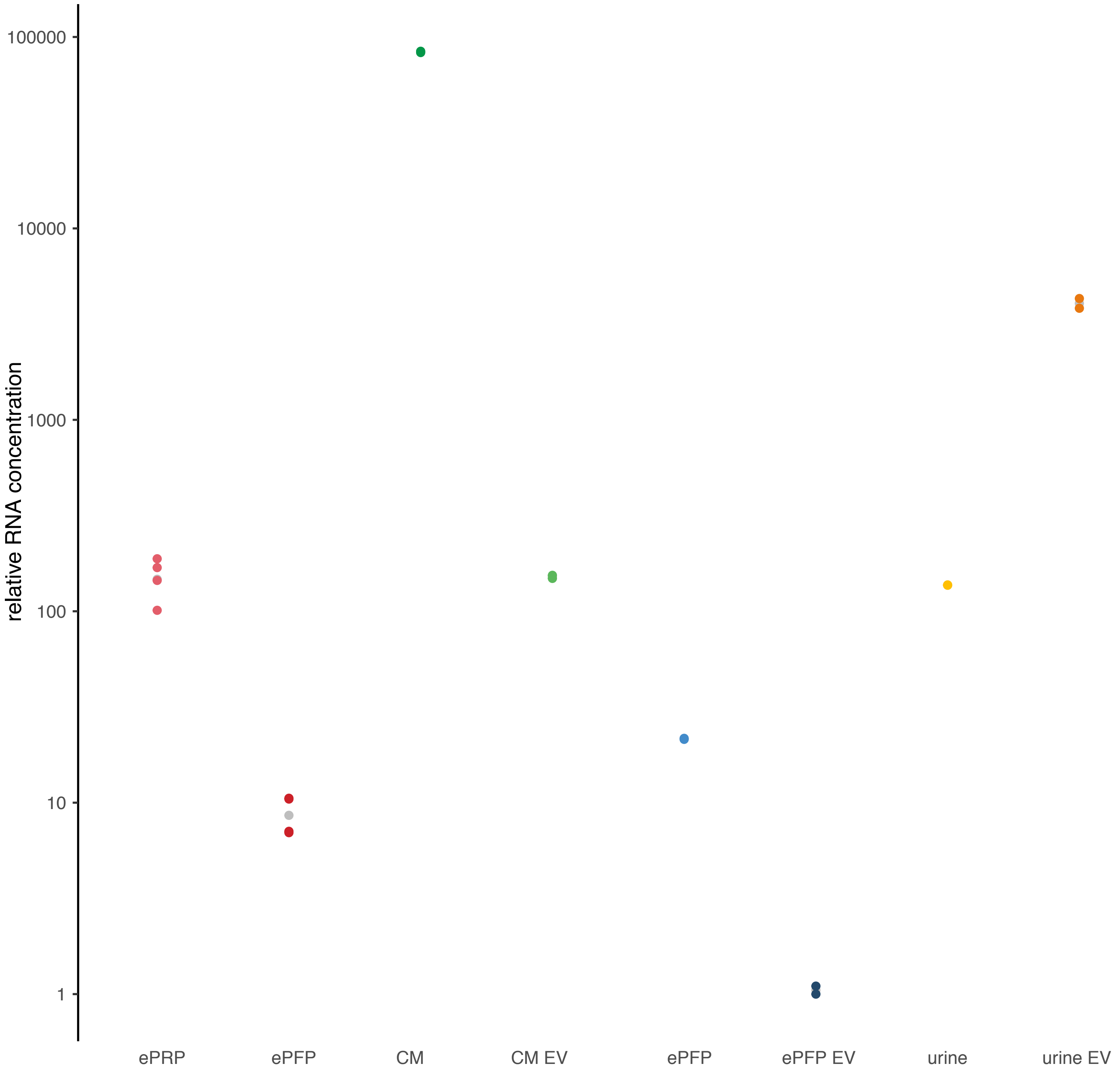

Supplemental Figure 6 Relative RNA concentration assessed by spike-in RNA (not corrected for original biofluid input volumes).

A

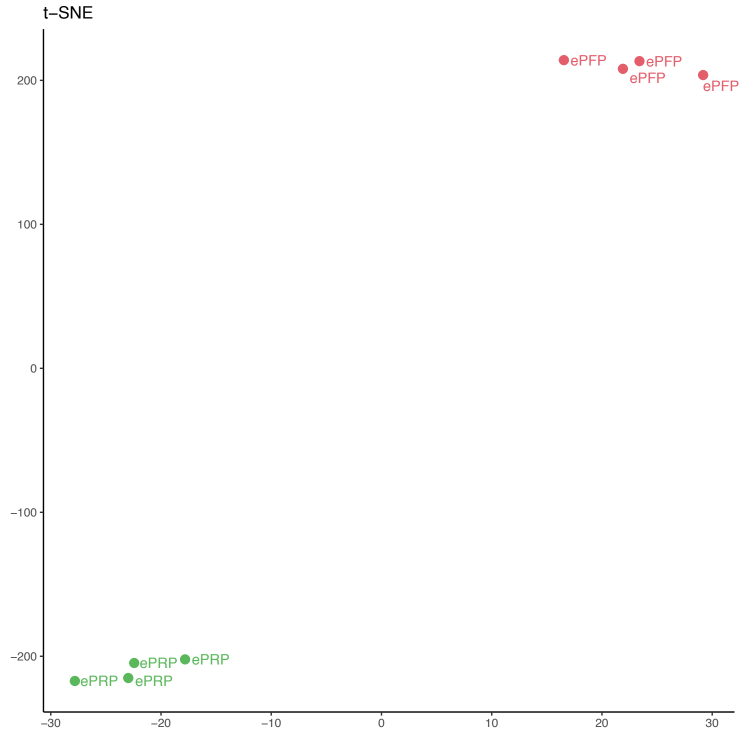

B

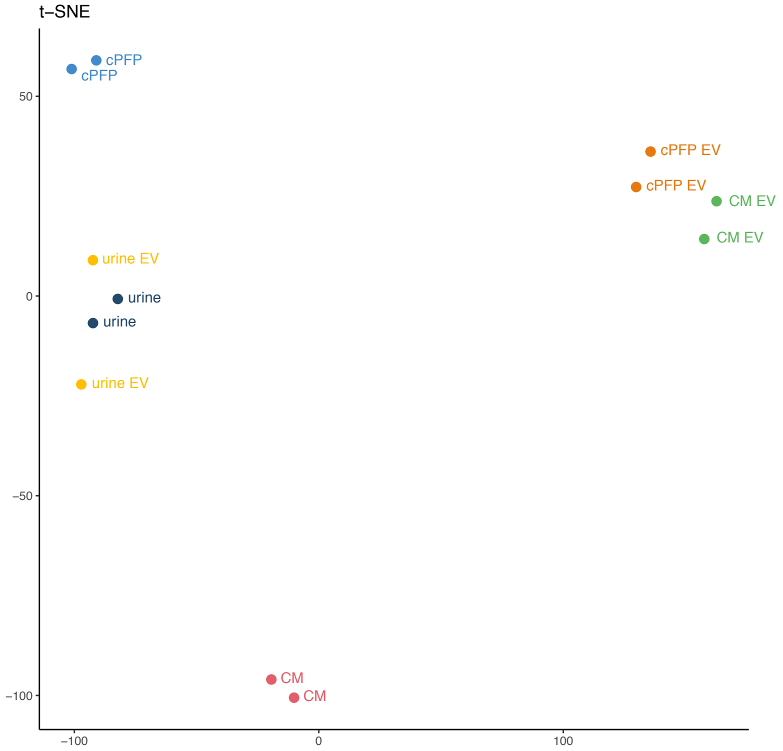

Supplemental Figure 7 t-SNE plots demonstrate the (dis)similarity of the sample biotypes.

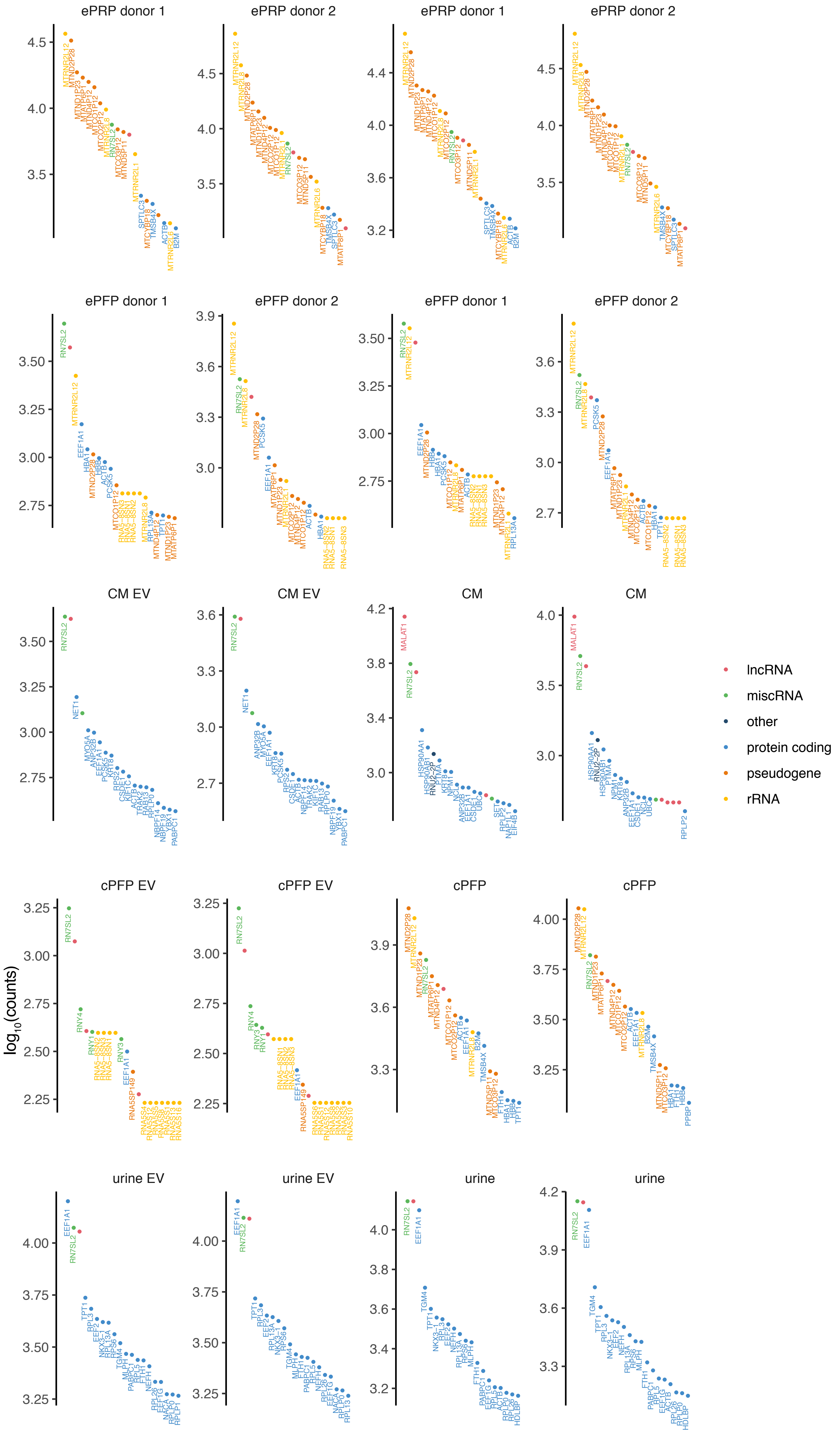

Supplemental Figure 8 Log_{10} counts of the 20 most abundant genes per sample.

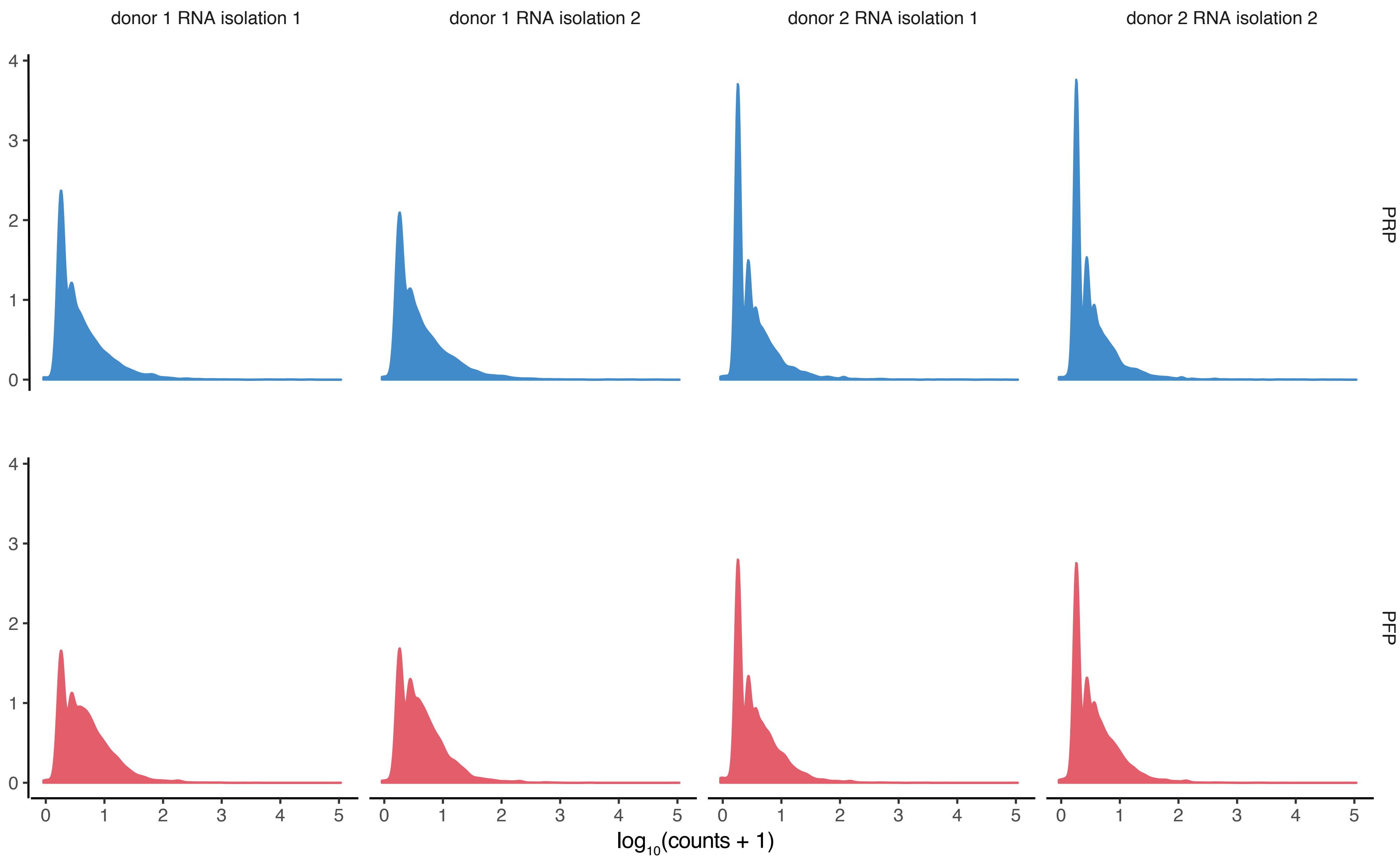

Supplemental Figure 9 Count distributions per sample.

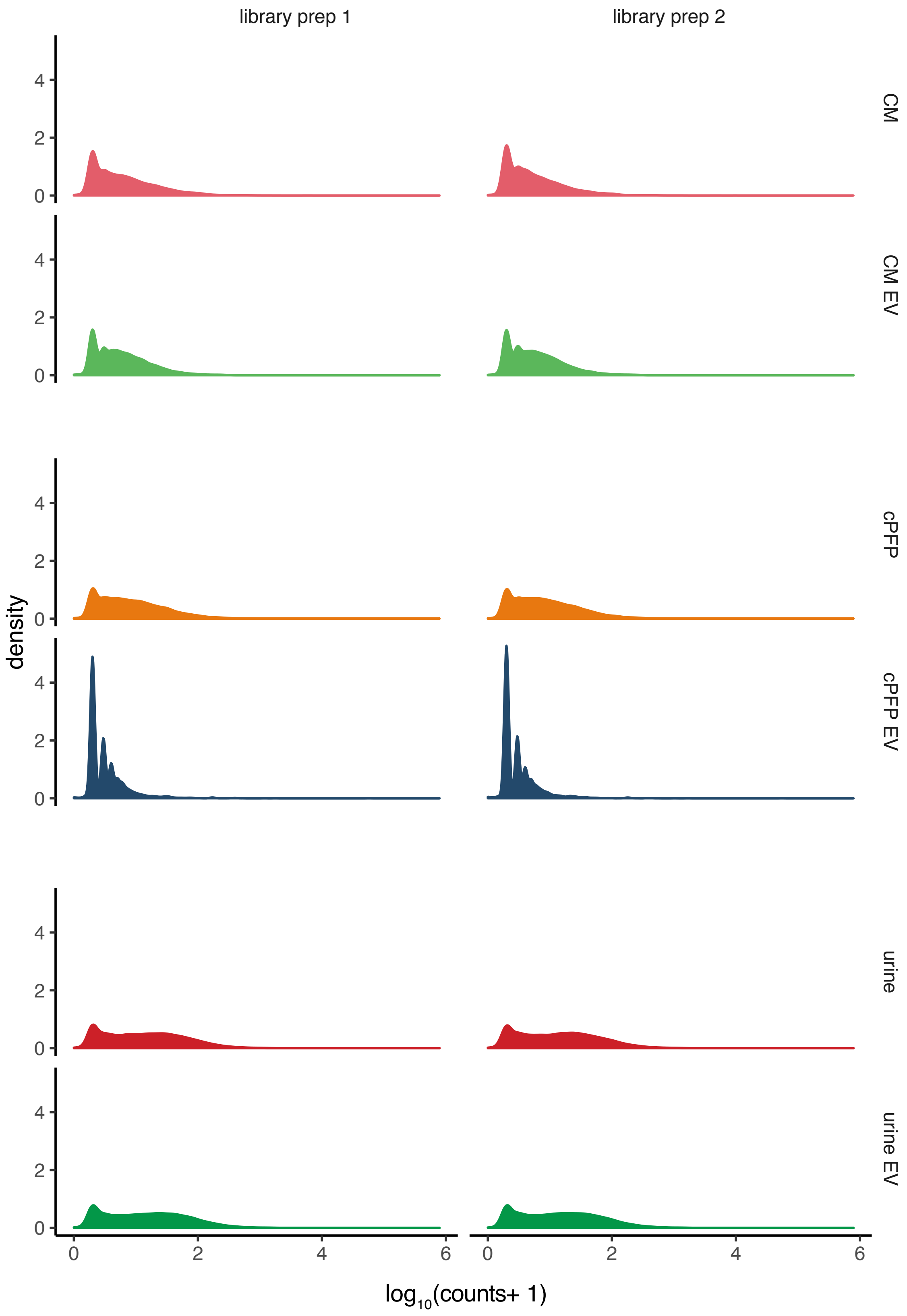

Supplemental Figure 9 Count distributions per sample.

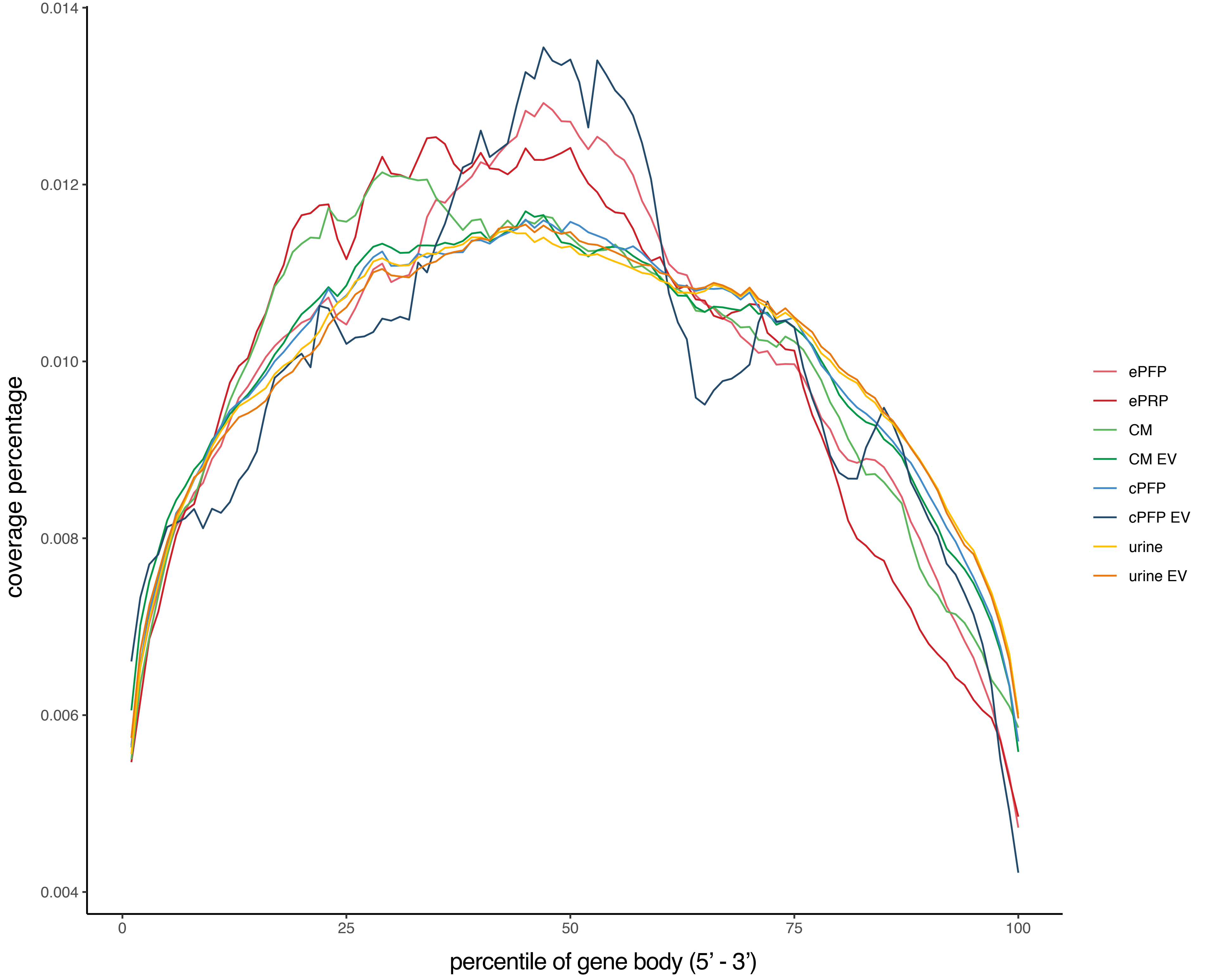

Supplemental Figure 10 Gene body coverage shows typical total RNA sequencing coverage of fragmented RNA.

A

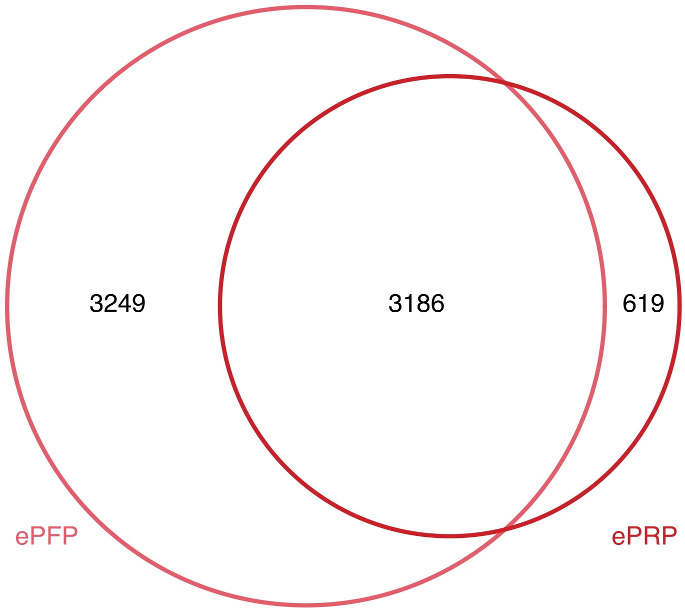

B

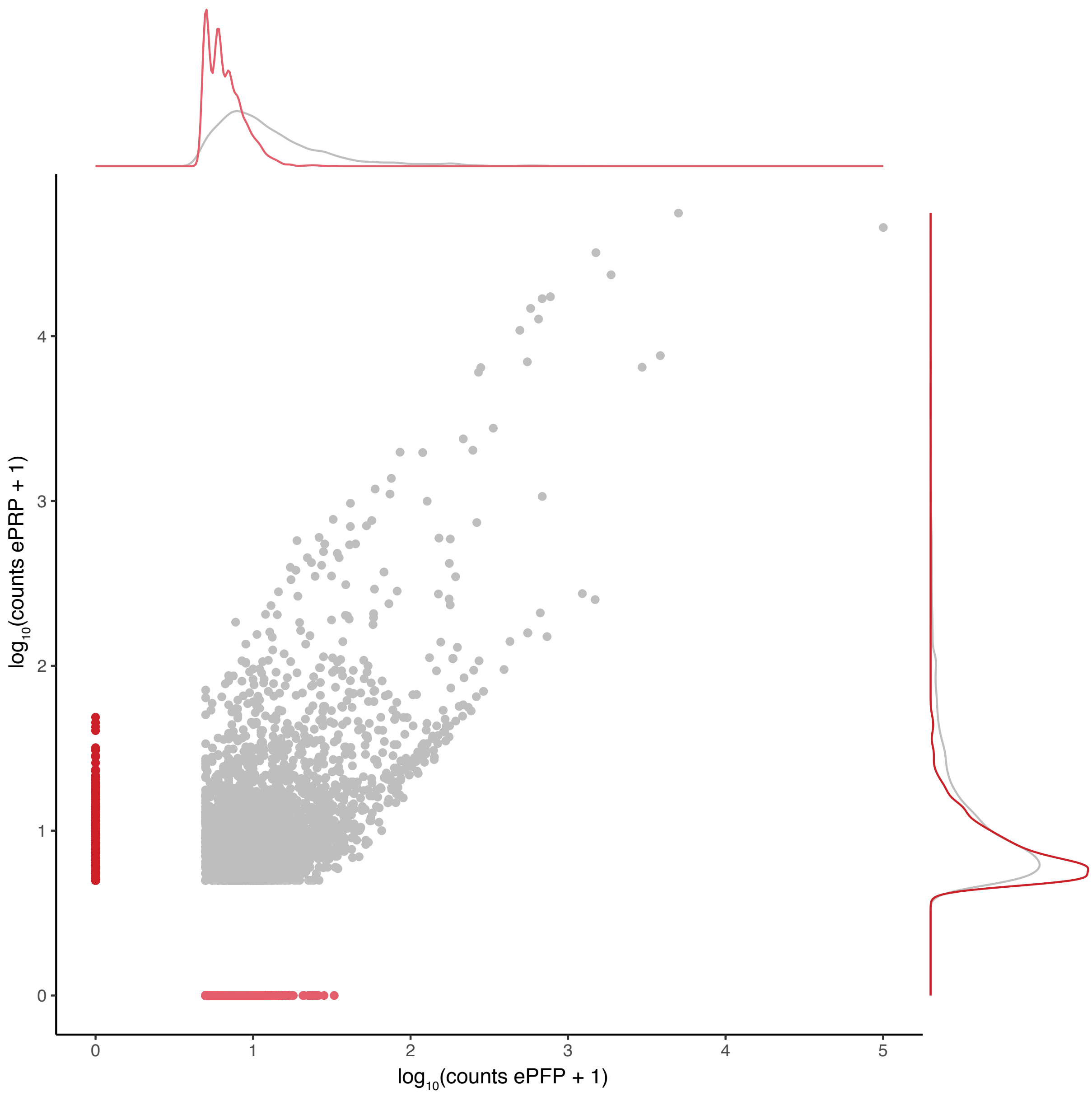

Supplemental Figure 11 Overlap of expressed genes for ePRP and ePFP. The ePRP unique genes show an equal distribution compared to the overlapping genes, while the ePFP unique genes are lower distributed.

Supplemental figure 2a (full-length blots):

Urine: ALIX, tsg101, CD9

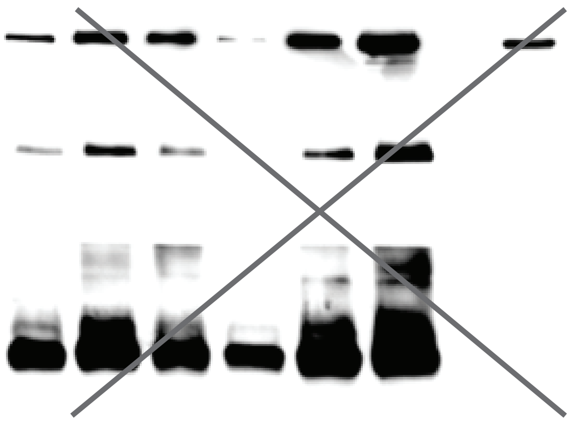

Plasma: flotilin-1, ApoA-1

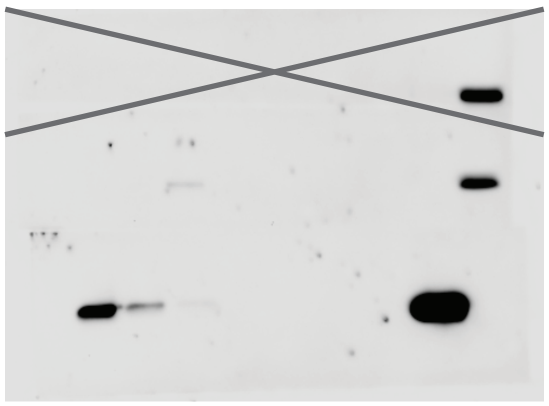

Urine: THP

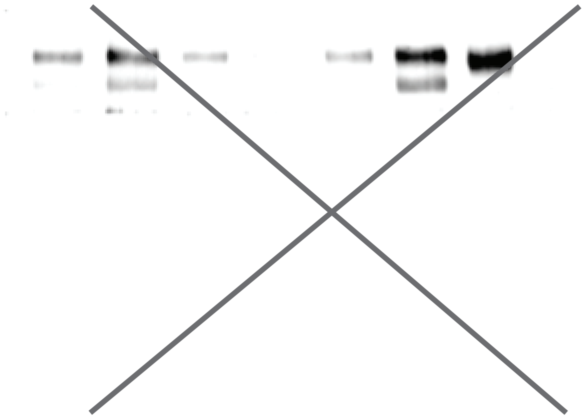

Plasma: CD9

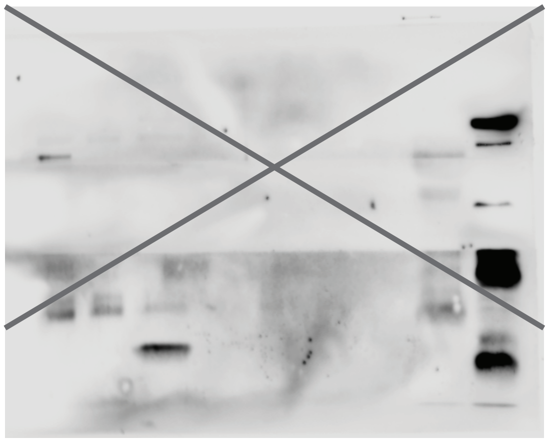

Supplemental figure 2b (full-length blots):

MCF-7 GFP-Rab27b: ALIX, tsg101, CD9

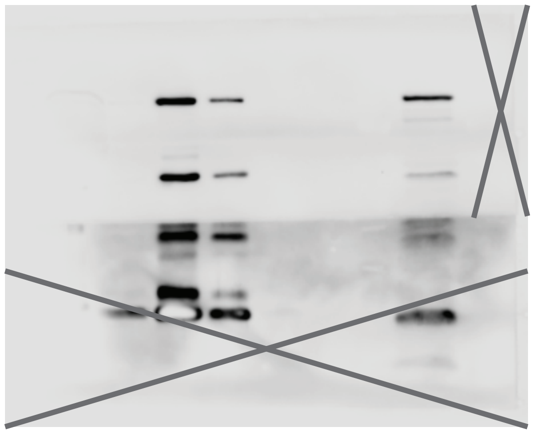

MCF-7 GFP-Rab27b: Ago2

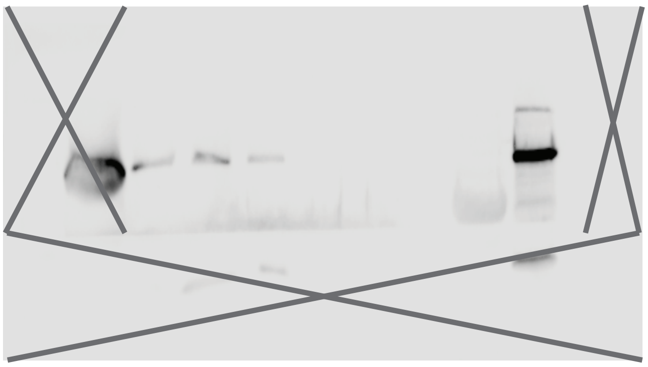

Supplemental Figure 12 Overview of the full-length blots used in supplemental figures 2a and 2b.
